# A Novel Point Set Registration-Based Hand–Eye Calibration Method for Robot-Assisted Surgery

**DOI:** 10.3390/s22218446

**Published:** 2022-11-03

**Authors:** Wenyuan Sun, Jihao Liu, Yuyun Zhao, Guoyan Zheng

**Affiliations:** Institute of Medical Robotics, School of Medical Engineering, Shanghai Jiao Tong University, Shanghai 200240, China

**Keywords:** hand–eye calibration, robot-assisted surgery, pedicle screw insertion, paired-point matching

## Abstract

Pedicle screw insertion with robot assistance dramatically improves surgical accuracy and safety when compared with manual implantation. In developing such a system, hand–eye calibration is an essential component that aims to determine the transformation between a position tracking and robot-arm systems. In this paper, we propose an effective hand–eye calibration method, namely registration-based hand–eye calibration (RHC), which estimates the calibration transformation via point set registration without the need to solve the AX=XB equation. Our hand–eye calibration method consists of tool-tip pivot calibrations in two-coordinate systems, in addition to paired-point matching, where the point pairs are generated via the steady movement of the robot arm in space. After calibration, our system allows for robot-assisted, image-guided pedicle screw insertion. Comprehensive experiments are conducted to verify the efficacy of the proposed hand–eye calibration method. A mean distance deviation of 0.70 mm and a mean angular deviation of 0.68° are achieved by our system when the proposed hand–eye calibration method is used. Further experiments on drilling trajectories are conducted on plastic vertebrae as well as pig vertebrae. A mean distance deviation of 1.01 mm and a mean angular deviation of 1.11° are observed when the drilled trajectories are compared with the planned trajectories on the pig vertebrae.

## 1. Introduction

Pedicle screw insertion is an effective treatment of spinal diseases, such as scoliosis, in addition to spinal fracture and vertebral injury. Manual implantation is challenging, especially in patients with severe spinal deformity, osteoporosis, or tumor [1,2,3]. To address the challenge, one of the proposed technologies is to integrate a robot arm with a computer navigation system [4,5,6,7]. In developing such a system, hand–eye calibration is an essential component, which aims to determine the homogeneous transformation between the robot hand/end-effector and the optical frame affixed to the end-effector [8,9].

Due to its importance, a number of approaches have been developed to solve the problem. Hand–eye calibration can be formulated in the form of AX=XB, where A and B are the robotic end-effector and the optical frame poses between successive time frames, respectively, and X is the unknown transformation matrix between the robot end-effector and the optical frame. Many solutions have been proposed to recover X-given data streams $\left\{A_{i}\right\}$ and $\left\{B_{i}\right\}$. Solutions to the problem can be roughly classified into four categories, i.e., separable solutions [10,11,12,13,14], simultaneous solutions [15,16,17], iterative solutions [8,18,19,20,21], and probabilistic methods [22,23]. Specifically, given equations A and B, it is possible to decompose the equation into rotational and translational parts. Separable solutions utilize this property to solve hand–eye calibration, where the rotation part is first solved, followed by solving the translational part. In contrast, simultaneous solutions solve the rotational and translational parts at the same time. Methods in the third category solve a nonlinear optimization problem by minimizing equations such as ||AX−XB||. As the algorithm iterates, it will converge on a solution to X. Different from methods of the first three categories, which assume an exact correspondence between the data streams $\left\{A_{i}\right\}$ and $\left\{B_{i}\right\}$, methods in the fourth category eliminate such a requirement.

Despite these efforts, accurate hand–eye calibration is challenging for the following reasons. First, although separable methods are useful, any error in the estimation for the rotational part is compounded when being applied to solving the translational part. Second, while simultaneous solutions can significantly reduce the propagation of error [24], they are sensitive to the nonlinearities present in measurements in the form of noise and errors [25]. Third, although it was observed that the nonlinear iterative approaches yielded better results to linear and closed-form solutions in terms of accuracy [25], they can be computationally expensive to carry out and may not always converge on the optimal solution.

In this paper, to tackle these challenges, we propose an effective hand–eye calibration method, namely registration-based hand–eye calibration (RHC), which estimates the calibration transformation via paired-point matching without the need to solve the AX=XB equation. Specifically, in our solution, we reformulate hand–eye calibration as tool-tip pivot calibrations in two-coordinate systems and a paired-point matching, taking advantage of the steady movement of the robot arm and thus reducing measurement errors and noise. The hand–eye calibration problem is then solved via closed-form solutions to three overdetermined equation systems. Our point set registration-based hand–eye calibration method has the following advantages:

- Our method is a simultaneous closed-form solution, which guarantees an optimal solution;
- Unlike other simultaneous solutions, our solution is obtained by solving three nonlinear least-square fitting problems, leading to three overdetermined equation systems. Thus, it is not sensitive to the nonlinearities present in measurements in the form of noise and errors;
- In comparison with the nonlinear iterative approaches, our method requires only simple matrix operations. Thus, it is computationally efficient;
- Our method achieves better results than the state-of-the-art (SOTA) methods.

The paper is organized as follows. Section 2 reviews related works. Section 3 presents the proposed method. Section 4 describes the experiments and results. Finally, we present discussions in Section 5, followed by our conclusion in Section 6.

## 2. Related Works

Huge amounts of time have been devoted to solve the problem of hand–eye calibration. Due to the wide applications of robot-assisted procedures, different types of methods have been developed for increased accuracy and robustness. Existing solutions can be roughly classified into four categories, as shown in Table 1, i.e., separable closed-form solutions [10,11,12,13,14], simultaneous closed-form solutions [15,16,17], iterative solutions [18,19,20], and probabilistic methods [22,23].

The earliest approaches separately estimated the rotational and translational parts. For example, Shiu et al. proposed a method for solving homogeneous transform equations [10]. Tsai presented an efficient 3D robotics hand–eye calibration algorithm that computed 3D position and orientation separably [11]. Quaternion-based [13], extrinsic hand–eye calibration [12], and dual-quaternions-based calibration methods [14] have been introduced for the individual estimations of rotational and translational parts. One known problem with separable methods is that any error in the estimation of the rotation matrices may be propagated to the estimation of the translation vector.

To avoid the error propagation problem with separable solutions, methods in the second category simultaneously compute the orientation and position. For example, Lu et al. proposed an approach that transformed the kinematic equation into linear systems using normalized quaternions [16]. Andreff et al. proposed an on-line hand–eye calibration method that derived a linear formulation of the problem [15]. Zhao et al. [17] proposed a hand–eye calibration method based on screw motion theory to establish linear equations and simultaneously solve rotation and translation. As confirmed by experimental results, simultaneous methods have less error than separable solutions [25].

Iterative solutions are another type of method used to solve the problem of error propagation. For example, Zhuang et al. [18] presented an iterative algorithm to solve the unknown matrix X in one stage, thus eliminating error propagation and improving noise sensitivity. Mao et al. [20] proposed using a direct linear closed-form solution followed by Jacobian optimization to solve AX=XB for hand–eye calibration. Hirsh et al. [26] proposed a robust iterative method to simultaneously estimate both the hand–eye and robot–world spatial transformation. Based on a metric defined on the group of the rigid transformation SE(3), Strobl and Hirzinger [27] presented an error model for nonlinear optimization. They then proposed a calibration method for estimating both the hand–eye and robot–world transformations. While iterative solutions are generally accurate, they can be computationally expensive and may not always converge to the optimal solution [28].

The methods mentioned above assume an exact correspondence between the streams of sensor data, while methods in the fourth category eliminate such a requirement. For example, Ma et al. [23] proposed two probabilistic approaches by giving new definitions of the mean on SE(3), which alleviated the restrictions on the dataset and led to improved accuracy. Although it is worth investigating the situation when the exact correspondence between sensor data is unknown, probabilistic methods usually lead to longer computation times. Additionally, assuming an exact correspondence is not a problem in our study.

Hand–eye calibration is also an active research topic in medical applications. For example, Morgan et al. [29] presented a Procrustean perspective-n-point (PnP) solution for hand–eye calibration for surgical cameras, achieving an average projection error of 12.99 pixels when evaluated on a surgical laparoscope. Özgüner et al. [30] proposed a solution for hand–eye calibration for the da Vinci robotic surgical system by breaking down the calibration procedure into systematic steps to reduce error accumulation. They reported a root mean square (RMS) error of 2.1 mm and a mean rotational error of 3.2 when their calibration method was used to produce visually-guided end-effector motions. Using the da Vinci Research Kit (dVRK) and an RGB-D camera, Roberti et al. [31] proposed to separate the calibration of the robotic arms and an endoscope camera manipulator from the hand–eye calibration of the camera for an improved accuracy in a 3D metric space. The proposed method reached a sub-millimeter accuracy in a dual-arm manipulation scenario, while the use of the RGB-D camera limited its actual application in surgery. Sun et al. [32] proposed a hand–eye calibration method for robot-assisted minimally invasive surgery, which relied purely on surgical instruments already in the operating scenario. Their model was formed by the geometry information of the surgical instrument and the remote center-of-motion (RCM) constraint, outperforming traditional hand–eye calibration methods in both simulation and robot experiments.

Deep learning-based methods, especially those based on convolutional neural networks (CNN), have also been developed for low-level image-processing tasks in hand–eye calibration [33,34,35,36]. For example, Valassakis et al. [34] proposed a sparse correspondence model that used a U-Net to detect 2D key points for eye-in-hand camera calibration. Kim et al. [36] introduced deep learning-based methods to restore out-of-focus blurred images for an improved accuracy in hand–eye calibration.

## 3. Materials and Methods

### 3.1. System Overview

Our robot-assisted, image-guided pedicle screw insertion system consists of a master computer, an optical tracking camera (Polaris Vega XT, NDI, Waterloo, ON, Canada) and a robot arm (UR 5e, Universal Robots, Odense, Denmark) with a guiding tube. The master computer communicates with the tracking camera to obtain poses of different optical tracking frames with the remote controller of the UR robot in order to realize a steady movement and to receive feedback information.

**Figure 1:**
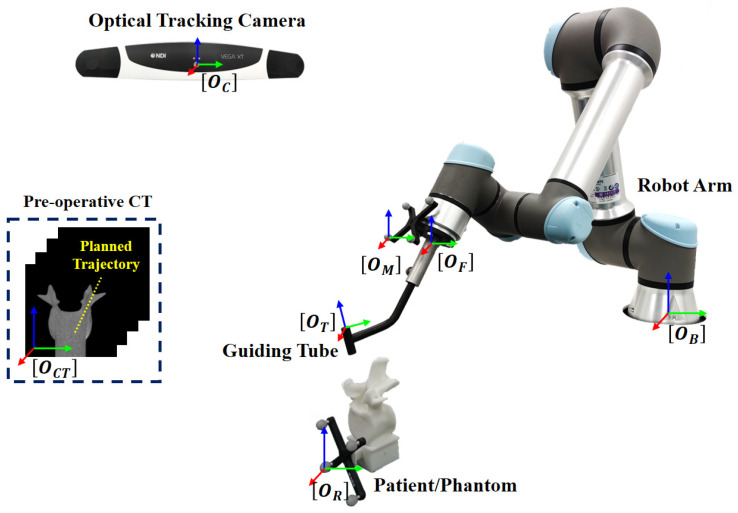
The involved coordinate systems for robot-assisted, image-guided pedicle screw insertion. During a pedicle screw insertion procedure, the pose of the guide is adjusted to align with a trajectory, which is planned in a pre-operative CT first, and then is transformed to the patient space via a surface registration.

During pedicle screw insertion, the target point and the aiming trajectory are planned in a pre-operative CT, which are transformed to the tracking camera space via a homogeneous transformation obtained by a surface registration [37]. Then, the pose of the guide will be adjusted to align with the planned trajectory. Thus, it is essential to determine the spatial transformation from the tracking camera space to the robot space, as shown in Figure 1. The transformation can be obtained via two different calibration procedures, including the hand–eye calibration and guiding tube calibration.

Our robot-assisted, image-guided pedicle screw insertion procedure involves the following coordinate systems (COS), as shown in Figure 1. The 3D COS of the optical tracking camera is represented by $O_{C}$; the 3D COS of the optical reference frame on the end-effector is represented by $O_{M}$; the 3D COS of the robotic flange is represented by $O_{F}$; the 3D COS of the guiding tube is represented by $O_{T}$; the 3D COS of the robot base is represented by $O_{B}$; the 3D COS of the pre-operative CT data is represented by $O_{CT}$; and the 3D COS of the optical reference frame attached to the patient/phantom is represented by $O_{R}$. At any time, poses of different optical tracking frames with respect to the tracking camera, such as ${}_{M}^{C}T$ and ${}_{R}^{C}T$, are known. At the same time, the pose of the robotic flange with respect to the robot base ${}_{F}^{B}T$ is known. This transformation information can be retrieved from the API (application programming interface) of the associated devices.

### 3.2. Registration-Based Hand–Eye Calibration

The aim of the hand–eye calibration is to establish the spatial transformation between the robot system and the optical tracking system. Mathematically, we solve the 4×4 spatial transformation matrix from the COS $O_{M}$ to the COS $O_{F}$, referred as ${}_{M}^{F}T$. In this subsection, the proposed registration-based hand–eye calibration (RHC) is introduced, which mainly consists of two steps: (1) solving tool-tip pivot calibrations in both the optical tracking camera COS $O_{C}$ and the robot base COS $O_{B}$; (2) solving hand–eye calibration via a paired-point matching.

#### 3.2.1. Tool-Tip Calibration

**Figure 2:**
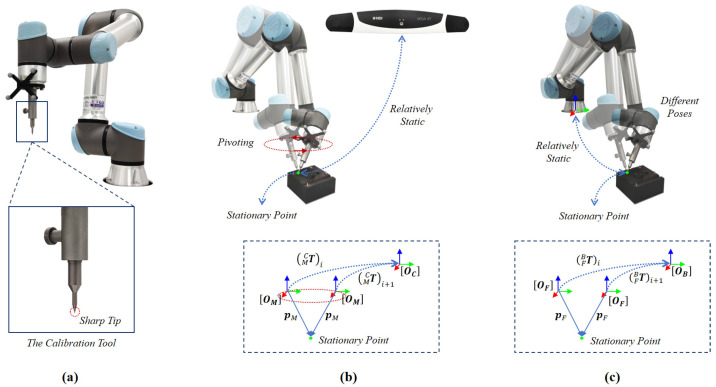
Tool-tip pivot calibration. (**a**) The calibration tool with a sharp tip is rigidly fixed to the flange during the hand–eye calibration; (**b**) pivot calibration of the offset of the tool tip with respect to the 3D COS $O_{M}$ of the optical reference frame on the end-effector; (**c**) pivot calibration of the offset of the tool tip with respect to the robotic flange COS $O_{F}$.

In the first step, we rigidly fixed a calibration tool with a sharp tip to the flange, as shown in Figure 2a. We then need to determine the coordinates of the tool tip relative to the respective two-coordinate systems, i.e., $O_{M}$ and $O_{F}$. We obtained both by pivot calibration [38]. Once calibrated, the coordinates of the tool tip with respect to $O_{M}$ and $O_{F}$ are known, which will then be used in the next step to compute a paired-point matching.

We will start to describe the pivot calibration of the coordinate of the tool tip with respect to the coordinate system $O_{M}$. We pivoted the tool tip around a stationary point, as shown in Figure 2b, to estimate the coordinates of the tool tip in both the optical tracking camera COS $O_{C}$ and the 3D COS $O_{M}$ of the optical reference frame on the end-effector. During pivoting, we placed the tool tip in a divot, which has the same size and shape with the tool tip to avoid any possible sliding. Then, we moved the tool around this pivot point while always touching the divot with its tip. We denoted, respectively, the two offsets as $p_{C}$ and $p_{M}$. During pivoting, we kept $p_{C}$ and $p_{M}$ static while collecting a set of *n* homogeneous transformations $\left\{{\left({}_{M}^{C}T\right)}_{i}=\left({\left({}_{M}^{C}R\right)}_{i},{\left({}_{M}^{C}t\right)}_{i}\right);1⩽i⩽n\right\}$ via the tracking camera API. Then, we estimated $p_{C}$ and $p_{M}$ by solving the following overdetermined equations:

(1) $$[\begin{array}{cc} {{(}_{M}^{C}R)}_{1} & -I\\ \vdots & \vdots \\ {{(}_{M}^{C}R)}_{n} & -I \end{array}] [\begin{array}{c} p_{M}\\ p_{C} \end{array}]= [\begin{array}{c} -{{(}_{M}^{C}t)}_{1}\\ \vdots \\ -{{(}_{M}^{C}t)}_{n} \end{array}]$$

where I is the 3×3 identity matrix.

Defining $\tilde{R}= [\begin{array}{cc} {\left({}_{M}^{C}R\right)}_{1} & -I\\ \vdots & \vdots \\ {\left({}_{M}^{C}R\right)}_{n} & -I \end{array}]$, and $\tilde{t}= [\begin{array}{c} -{\left({}_{M}^{C}t\right)}_{1}\\ \vdots \\ -{\left({}_{M}^{C}t\right)}_{n} \end{array}]$, we have:

(2) $$\tilde{R} [\begin{array}{c} p_{M}\\ p_{C} \end{array}]=\tilde{t}$$

Then, we can solve $p_{M}$ and $p_{C}$ using pseudo-inverse [39]:

(3) $$[\begin{array}{c} p_{M}\\ p_{C} \end{array}]={\left({\left(\tilde{R}\right)}^{T}\left(\tilde{R}\right)\right)}^{-1}{\left(\tilde{R}\right)}^{T}\tilde{t}$$

As we are only interested in knowing the offset of the tool tip with respect to $O_{M}$, we keep $p_{M}$ while disregarding $p_{C}$.

Similarly, we can use the same pivot calibration technique to estimate the coordinates of the tool tip in both the robotic flange COS $O_{F}$ and the robot base COS $O_{B}$. This time, we pivoted the tool tip around a stationary point, as shown in Figure 2c. Similarly, we placed the tool tip in a divot to avoid sliding. We denoted, respectively, the two coordinates as $p_{B}$ and $p_{F}$. During pivoting, we kept $p_{B}$ and $p_{F}$ static while collecting a set of *l* homogeneous transformations $\left\{{\left({}_{F}^{B}T\right)}_{i}=\left({\left({}_{F}^{B}R\right)}_{i},{\left({}_{F}^{B}t\right)}_{i}\right);1⩽i⩽l\right\}$ via the robot arm API. Then, we estimated $p_{B}$ and $p_{F}$ by solving following the following overdetermined equations [39]:

(4) $$[\begin{array}{cc} {{(}_{F}^{B}R)}_{1} & -I\\ \vdots & \vdots \\ {{(}_{F}^{B}R)}_{l} & -I \end{array}] [\begin{array}{c} p_{F}\\ p_{B} \end{array}]= [\begin{array}{c} -{{(}_{F}^{B}t)}_{1}\\ \vdots \\ -{{(}_{F}^{B}t)}_{l} \end{array}]$$

where I is the 3×3 identity matrix.

Again, we are only interested in knowing the offset of the tool tip with respect to $O_{F}$; therefore, we kept $p_{F}$ while disregarding $p_{B}$.

#### 3.2.2. Solving Hand–Eye Calibration via Paired-Point Matching

**Figure 3:**
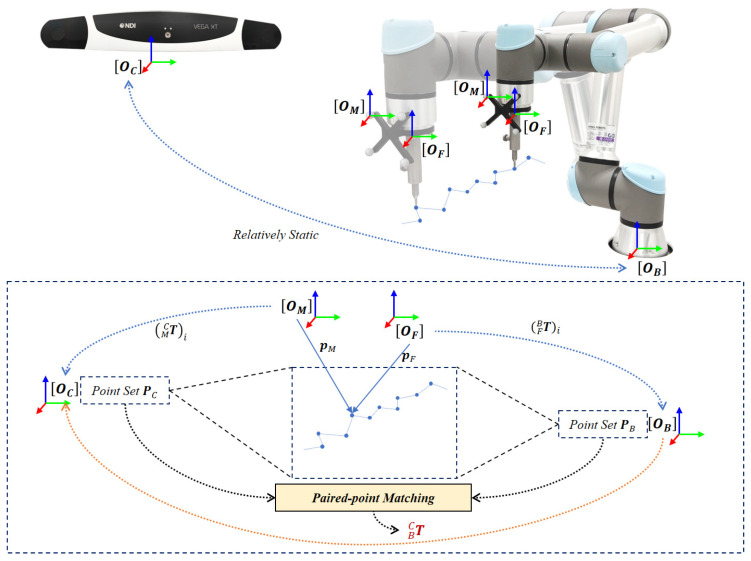
Solving ${}_{B}^{C}T$ via paired-point matching. By controlling the flange to move in *m* different positions, we can obtain the coordinates of the tool tip in both the optical tracking camera COS $O_{C}$ and the robot base COS $O_{B}$, generating two point sets. ${}_{B}^{C}T$ is solved by matching the two point sets using a paired-point matching algorithm.

After obtaining the offsets of the tool tip with respect to two-coordinate systems $O_{M}$ and $O_{F}$, we can compute the coordinates of the tool tip in both the tracking camera COS $O_{C}$ and the robot base COS $O_{B}$ at any time via the corresponding device’s API. In this section, we present an elegant method to solve the hand–eye calibration via paired-point matching using the setup shown in Figure 3.

Specifically, during the hand–eye calibration, we maintained a stationary spatial relationship between the robot base and the tracking camera while moving the robot flange. By controlling the flange to move in *m* different positions, we collected two set of points $P_{C}= [\begin{array}{ccc} {\left(p_{C}\right)}_{1} & \cdots & {\left(p_{C}\right)}_{m} \end{array}]$, which are the coordinates of the tool tip measured in the tracking camera COS $O_{C}$ via ${\left(p_{C}\right)}_{i}={\left({}_{M}^{C}T\right)}_{i}p_{M}$ and $P_{B}= [\begin{array}{ccc} {\left(p_{B}\right)}_{1} & \cdots & {\left(p_{B}\right)}_{m} \end{array}]$, which are the coordinates of the tool tip measured in the robot base COS $O_{B}$ via ${\left(p_{B}\right)}_{i}={\left({}_{F}^{B}T\right)}_{i}p_{F}$. Therefore, we can solve the spatial transformation ${}_{B}^{C}T$ using a paired-point matching algorithm.

For the first step to match two paired-point sets, we computed a 3×3 matrix H as follows:

(5) $$H=\sum_{j=1}^{m}({\left(p_{B}\right)}_{j}-(\frac{1}{m}\sum_{i=1}^{m}{\left(p_{B}\right)}_{i}))\cdot ({\left(p_{C}\right)}_{j}-(\frac{1}{m}\sum_{i=1}^{m}{\left(p_{C}\right)}_{i}){)}^{T}$$

We then used the singular value decomposition (SVD) [39] to decompose matrix H into U, S, and V matrices:

(6) $$H=USV^{T}$$

Based on the decomposed matrices, we computed the rotation matrix ${}_{B}^{C}R$ as:

(7) $${}_{B}^{C}R=V [\begin{array}{ccc} 1 & 0 & 0\\ 0 & 1 & 0\\ 0 & 0 & \lambda \end{array}]U^{T}$$

where λ=det(UV).

Based on ${}_{B}^{C}R$, we solved ${}_{B}^{C}t$ using:

(8) $${}_{B}^{C}t=\frac{1}{m}\sum_{i=1}^{m}{\left(p_{C}\right)}_{i}-{}_{B}^{C}R\frac{1}{m}\sum_{i=1}^{m}{\left(p_{B}\right)}_{i}$$

Therefore, we obtained the spatial transformation ${}_{B}^{C}T$ as:

(9) $${}_{B}^{C}T=\left({}_{B}^{C}R,{}_{B}^{C}t\right)$$

For each position in the movement trajectory, we computed the spatial transformation ${{(}_{M}^{F}T)}_{i}$ as:

(10) $${{(}_{M}^{F}T)}_{i}={{(}_{F}^{B}T)}_{i}^{-1}\cdot {{(}_{B}^{C}T)}^{-1}\cdot {{(}_{M}^{C}T)}_{i}$$

where ${{(}_{F}^{B}T)}_{i}$ and ${{(}_{M}^{C}T)}_{i}$ are retrieved from the associated device’s API when generating $P_{C}$ and $P_{B}$.

Each position will give a different ${{(}_{M}^{F}T)}_{i}$. To improve the robustness and to increase the accuracy, we averaged all the obtained transformations. Specifically, we used $\left(\psi_{i},\theta_{i},\phi_{i}\right)$ to represent the Euler angles of ${{(}_{M}^{F}R)}_{i}$, so the average rotation matrix ${}_{M}^{F}R$ can be written as:

(11) $${}_{M}^{F}\bar{R}=R\left(\frac{1}{m}\sum_{i=1}^{m}\psi_{i},\frac{1}{m}\sum_{i=1}^{m}\theta_{i},\frac{1}{m}\sum_{i=1}^{m}\phi_{i}\right)$$

where R() represents the transformation from the Euler angles to the rotation matrix. Meanwhile, the average translation vector ${}_{M}^{F}t$ can be written as:

(12) $${}_{M}^{F}\bar{t}=\frac{1}{m}\sum_{i=1}^{m}{{(}_{M}^{F}t)}_{i}$$

where ${{(}_{M}^{F}t)}_{i}$ is the translation vector of ${{(}_{M}^{F}T)}_{i}$.

Therefore, the hand–eye transformation ${}_{M}^{F}T$ is composed of the average rotation matrix ${}_{M}^{F}\bar{R}$ and average translation vector ${}_{M}^{F}\bar{t}$, written as:

(13) $${}_{M}^{F}T={(}_{M}^{F}\bar{R}{,}_{M}^{F}\bar{t})$$

### 3.3. Guiding Tube Calibration

**Figure 4:**
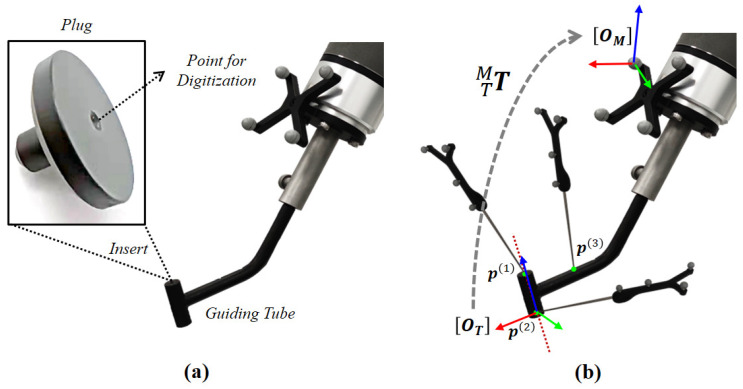
A schematic view the guiding tube calibration. (**a**) The plug, which can be inserted into the guiding tube from both ends for digitization. (**b**) The three points on the tube that are digitized and the COS $O_{T}$ of the guiding tube established using the three points.

To achieve the robot-assisted pedicle screw insertion, the guiding tube that guides the drilling of a screw insertion trajectory needs to be calibrated. The guiding tube calibration is a procedure to estimate the transformation ${}_{M}^{T}T$ of the COS $O_{T}$ defined on the guiding tube relative to the COS $O_{M}$ of the optical reference frame attached to the robot end-effector. In this calibration procedure, we utilized two COSs, i.e., the local COS $O_{T}$ of the guiding tube and the COS $O_{M}$, as shown in Figure 4.

The local COS $O_{T}$ can be determined using three points: the two end points of the guiding tube that lie on the center axis of the tube (referred as $p^{\left(1\right)}$ and $p^{\left(2\right)}$), and one further point that is on the guiding tube (referred as $p^{\left(3\right)}$). To digitize $p^{\left(1\right)}$ and $p^{\left(2\right)}$, we used a plug to insert into the guiding tube. We then digitized the coordinates of these three points in the COS $O_{M}$, referred as $p_{M}^{\left(1\right)}$, $p_{M}^{\left(2\right)}$, and $p_{M}^{\left(3\right)}$, respectively.

To establish the COS $O_{T}$, we defined the origin by $p^{\left(2\right)}$, the *z*-axis by $p^{\left(1\right)}$ and $p^{\left(2\right)}$, and determined the three points by the *x*-*z* plane. We obtained the transformation ${}_{M}^{T}T$ by its origin and axes, as:

(14) $${}_{T}^{M}T= [\begin{array}{cccc} \frac{r_{M}^{\left(x\right)}}{∥r_{M}^{\left(x\right)}∥} & \frac{r_{M}^{\left(y\right)}}{∥r_{M}^{\left(y\right)}∥} & \frac{r_{M}^{\left(z\right)}}{∥r_{M}^{\left(z\right)}∥} & p_{M}^{\left(2\right)}\;\\ 0 & 0 & 0 & 1 \end{array}]$$

where,

(15) $$\left\{\begin{array}{l} r_{M}^{\left(x\right)}=\left(\left(p_{M}^{\left(3\right)}-p_{M}^{\left(2\right)}\right)\times \left(p_{M}^{\left(1\right)}-p_{M}^{\left(2\right)}\right)\right)\times \left(p_{M}^{\left(1\right)}-p_{M}^{\left(2\right)}\right)\;\\ r_{M}^{\left(y\right)}=\left(p_{M}^{\left(3\right)}-p_{M}^{\left(2\right)}\right)\times \left(p_{M}^{\left(1\right)}-p_{M}^{\left(2\right)}\right)\;\\ r_{M}^{\left(z\right)}=p_{M}^{\left(1\right)}-p_{M}^{\left(2\right)} \end{array}\right.$$

### 3.4. Robot-Assisted Pedicle Screw Insertion

**Figure 5:**
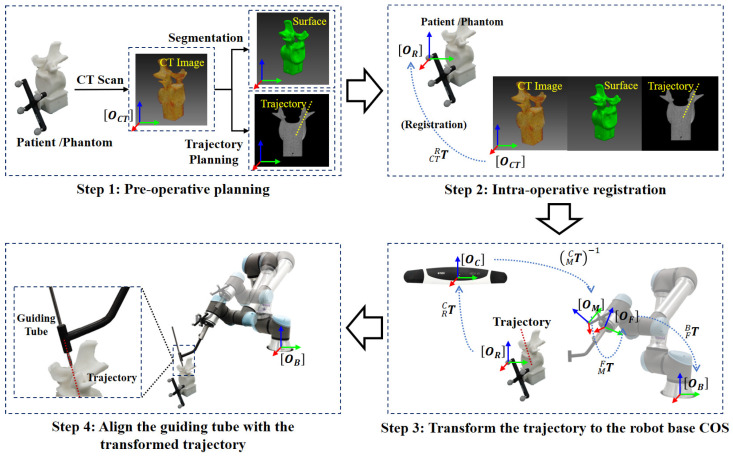
A schematic view of robot-assisted pedicle screw insertion: The target trajectory is planned in the COS $O_{CT}$ and transformed to the COS $O_{R}$ by intra-operative registration. The target trajectory is further transformed to the robot base COS $O_{B}$. The guiding tube is aligned with the target trajectory for insertion guidance.

Figure 5 illustrates the schematic view of the robot-assisted pedicle screw insertion procedure. The workflow of the robot-assisted, image-guided pedicle screw insertion consists of following three steps: (1) pre-operative trajectory planning; (2) intra-operative registration; (3) transforming the planned trajectory to the robot base COS $O_{B}$ and aligning the guiding tube with the transformed trajectory.

#### 3.4.1. Pre-Operative Planning

In the first step, we obtained a pre-operative CT scan before the operation. We segmented the target vertebra in the CT image and defined a trajectory using an entry point $p_{CT}^{\left(e\right)}$ and a target point $p_{CT}^{\left(t\right)}$ in the image space.

#### 3.4.2. Intra-Operative Registration

In the second step, we performed an intra-operative registration to establish the spatial transformation ${}_{CT}^{R}T$ from the CT image COS $O_{CT}$ to the COS $O_{R}$. By digitizing points on the surface, we adopted a surface registration algorithm [37] to solve ${}_{CT}^{R}T$. Based on ${}_{CT}^{R}T$, $p_{CT}^{\left(e\right)}$ and $p_{CT}^{\left(t\right)}$ can be transformed to the COS $O_{R}$.

#### 3.4.3. Transforming the Planned Trajectory to the Robot Base COS and Aligning the Guiding Tube with the Transformed Trajectory

In the third step, we transformed the planned trajectory to the robot base COS $O_{B}$ so that the robot can align the guiding tube with the transformed trajectory, which is calculated as:

(16) $$[\begin{array}{cc} p_{B}^{\left(e\right)} & p_{B}^{\left(t\right)} \end{array}]={}_{F}^{B}T\cdot {}_{M}^{F}T\cdot {\left({}_{M}^{C}T\right)}^{-1}\cdot {}_{R}^{C}T\cdot {}_{CT}^{R}T [\begin{array}{cc} p_{CT}^{\left(e\right)} & p_{CT}^{\left(t\right)} \end{array}]$$

In Equation (16), we retrieved ${}_{R}^{C}T$ and ${}_{M}^{C}T$ from the optical tracking camera’s API. ${}_{M}^{F}T$ is the hand–eye transformation. We retrieved ${}_{F}^{B}T$ from the robot arm’s API.

## 4. Experiments and Results

In this section, we will introduce the experiments and results of our study. We designed and conducted three experiments to investigate the efficacy of the proposed method: (1) an investigation of the influence of the range of robot movement to hand–eye calibration; (2) a comparison with state-of-the-art hand–eye calibration methods; and (3) an overall system accuracy study.

### 4.1. Metrics

**Figure 6:**
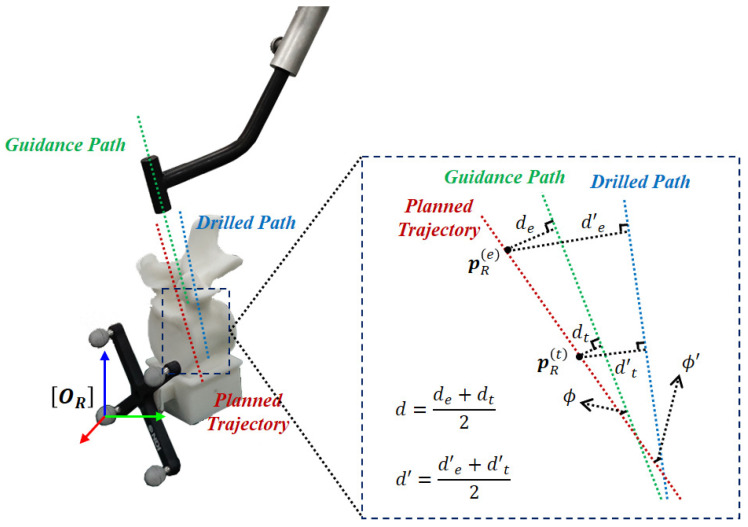
Metrics used to evaluate the accuracy in this study, including distance deviation *d* and $d^{\prime }$, as well as incline angle ϕ and $\phi^{\prime }$.

In the experiments, the performance is quantified by the deviations between the actual path and the planned trajectory. The deviations consist of the incline angle (unit: ) and distance deviation (unit: mm). We used the entry point $p^{\left(e\right)}$ and the target point $p^{\left(t\right)}$ on the planned trajectory to measure the distance, as shown in Figure 6. The distance deviation and incline angle between the guidance path and the planned trajectory are denoted as *d* and ϕ, respectively, while the distance deviation and the incline angle between the drilled path and the planned trajectory are denoted as $d^{\prime }$ and $\phi^{\prime }$, respectively.

### 4.2. Investigation of the Influence of the Range of Robot Movement to the Hand–Eye Calibration

**Figure 7:**
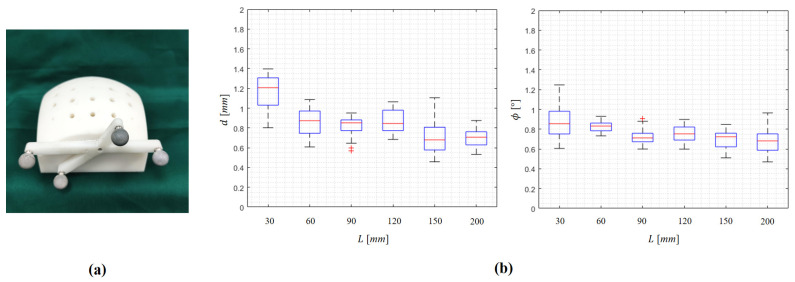
Investigation of the influence of the range of robot movement to the hand–eye calibration: (**a**) the plastic phantom used in the experiment; (**b**) the box plots of distance deviation and incline angle.

In this experiment, we investigated the influence of the spatial range of robot movement to the proposed RHC. In the experiment, a plastic phantom was designed and used, as shown in Figure 7a. The phantom, which was fabricated by 3D printing, had a dimension of 140×90× 85 mm${}^{3}$, and 25 trajectories were planned on the phantom.

During the hand–eye calibration, the robot is controlled to move in an L×L×L mm${}^{3}$ cubic space. To investigate the influence of the range of robot movement, we calibrated different hand–eye transformation matrices with an *L* of 30, 60, 90, 120, 150, or 200 mm. Each time, after obtaining hand–eye calibration, we used the obtained transformation to control the robot to align the guiding tube with a planned trajectory. After that, we digitized the guidance path to evaluate the alignment accuracy.

Experimental results are shown in Figure 7 and Table 2. Both *d* and ϕ decreased when *L* increased. When *L* was 200 mm, the mean distance deviation was 0.70 mm and the mean incline angle was 0.68. The results demonstrate that the larger the robot movement range, the higher the hand–eye calibration accuracy. However, further increasing the movement range will lead to a failure in tracking by the camera. We found that the maximally allowed robot movement range is 200×200× 200 mm${}^{3}$.

### 4.3. Comparison with State-of-the-Art Hand–Eye Calibration Methods

The plastic phantom introduced in Section 4.2 was also used in this study to compare our method with state-of-the-art (SOTA) hand–eye calibration methods, including Tsai’s method [11], Andreff’s method [15], Chou’s method [13], Shah’s method [40], and Wu’s method [8]. Each time, after obtaining hand–eye calibration using one of the mentioned methods, we used the obtained transformation to control the robot to align the guiding tube with a planned trajectory. After that, we then digitized the guidance path to evaluate the alignment accuracy, which reflects the hand–eye calibration accuracy.

**Figure 8:**
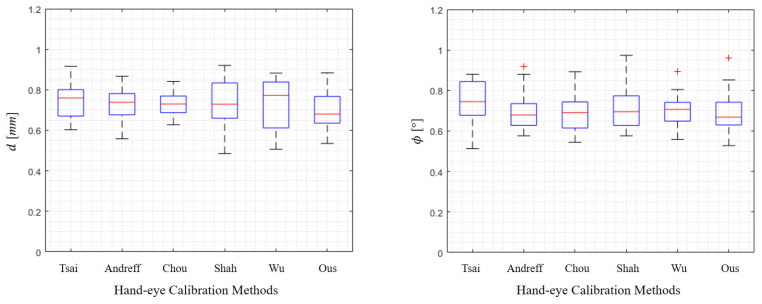
Comparison of our method with the SOTA hand–eye calibration methods.

The distance deviation *d* and the angular deviation ϕ are shown in Figure 8 and Table 3. We also report the computational time cost for each method in Table 3. In comparison with the SOTA methods, our method achieved the best results in terms of distance deviation and incline angle. Meanwhile, the time cost of our method is much lower than the iterative calibration method [8], as shown in Table 3.

### 4.4. Overall System Accuracy Study

To evaluate the overall system accuracy, we conducted trajectory drilling experiments on three types of objects: (a) the plastic phantom used in Section 4.2, (b) four 3D-printed vertebrae, and (c) eight pig vertebrae. Each time, we controlled the robot to align the guiding tube with the planned trajectory and drilled a trajectory into the test subject. In total, we planned and drilled 20 trajectories on the plastic phantom, another 8 trajectories on the 3D-printed vertebrae and further another 8 trajectories on the pig vertebrae. For each trajectory, after drilling, both the guidance paths and the drilled paths were digitized to measure the accuracy.

**Figure 9:**
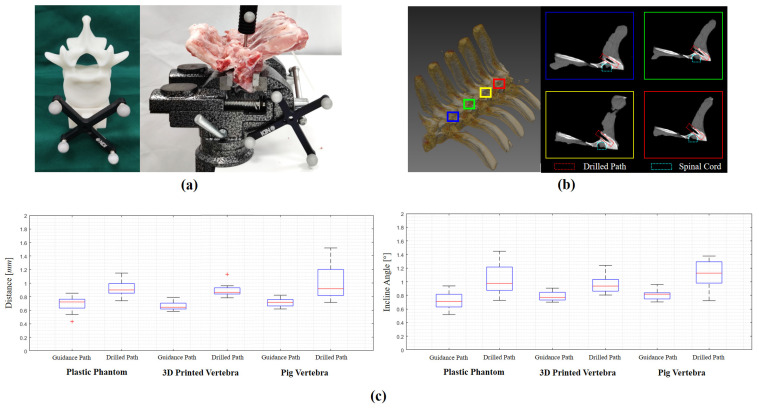
Overall system accuracy study: (**a**) the 3D-printed vertebra and pig vertebrae; (**b**) the CT image of the animal vertebrae after drilling; (**c**) the box plots of distance deviation and incline angle.

Results are shown in Figure 9 and Table 4. Specifically, on the plastic phantom, the average distance deviation and the average incline angle between the guiding paths and the planned trajectories are 0.70 mm and 0.72°, respectively, while the average distance deviation and the average incline angle between the drilled trajectories and the planned trajectories are 0.93 mm and 1.04°, respectively. Additionally, on the 3D-printed vertebrae, our system achieved a slightly better result, i.e., the average distance deviation and the average incline angle between the guiding paths and the planned trajectories are 0.66 mm and 0.79, respectively, and the average distance deviation and the average incline angle between the drilled trajectories and the planned trajectories are 0.90 mm and 0.96°, respectively. Finally, we evaluated our system accuracy on the pig vertebrae. The average distance deviation and the average incline angle between the guiding paths and the planned trajectories are 0.71 mm and 0.82°, respectively, while the average distance deviation and the average incline angle between the drilled trajectories and the planned trajectories are 1.01 mm and 1.11°, respectively. Figure 9b shows a post-operative CT scan of the drilled path on a pig vertebra, demonstrating the high accuracy of our system. Both quantitative and qualitative results demonstrate that our system accuracy is good enough for robot-assisted pedicle screw insertion.

## 5. Discussions

Hand–eye calibration is one of the essential components when developing a robot-assisted, image-guided pedicle screw insertion system. The accuracy of hand–eye calibration will have a direct influence on the system accuracy. However, it is challenging to develop an accurate and robust method for hand–eye calibration. In this paper, we proposed an effective hand–eye calibration method based on tool-tip pivot calibration and paired-point matching without the need to solve the AX=XB equation. Comprehensive experiments were conducted to validate the accuracy of our proposed hand–eye calibration method as well as the robot-assisted, image-guided pedicle screw insertion system. Both qualitative and quantitative results demonstrate the efficacy of our hand–eye calibration method and the high accuracy of our system.

In comparison with a SOTA hand–eye calibration method, our method has the following advantages: First, our method is a simultaneous closed-form solution, which is derived by solving three overdetermined equations, guaranteeing an optimal solution. Second, unlike other simultaneous solutions, we reformulate the hand–eye calibration problem as solutions to tool-tip pivot calibrations in two-coordinate systems and paired-point matching, taking advantage of the steady movement of the robot arm, thus reducing measurement errors and noise. Third, in comparison with methods depending on iterative solutions [18,19,20,21] or probabilistic models [22,23], our method is much faster because it is not an iterative solution and only requires simple matrix operations.

Based on the novel hand–eye calibration method, we further developed a robot-assisted, image-guided pedicle screw insertion system. We conducted trajectory drilling experiments on a plastic phantom, 3D-printed vertebrae, and pig vertebrae to validate the accuracy of our system. When drilling trajectories on the plastic phantom, our system achieved a mean distance deviation of 0.93 mm and a mean angular deviation of 1.04°. When it was used to drill trajectories on the 3D-printed vertebrae, our system achieved a mean distance deviation of 0.90 mm and a mean angular deviation of 0.96°. To check whether the differences between results obtained from the plastic phantom and the 3D-printed vertebrae are statistically significant, we conducted an unpaired t-test and chose a significant level of α=0.05. We found a *p*-value of 0.52 for the distance deviation and a *p*-value of 0.40 for the angular deviation. When drilling trajectories on the pig vertebrae, our system achieved a mean distance deviation of 1.01 mm and a mean angular deviation of 1.11°, which are regarded accurate enough for pedicle screw insertion.

## 6. Conclusions

In this paper, we proposed a novel hand–eye calibration method, namely registration-based hand–eye calibration (RHC), to estimate the calibration transformation via paired-point matching without the need to solve the AX=XB equation. Based on the proposed hand–eye calibration method, we developed a robot-assisted, image-guided pedicle screw insertion system. Comprehensive experiments were conducted to investigate the influence of the range of robot movement on the hand–eye calibration to compare our method with state-of-the-art methods and to evaluate overall system accuracy. Our experimental results demonstrate the efficacy of our hand–eye calibration method and the high accuracy of our system. Our novel hand–eye calibration method can be applied to other types of robot-assisted surgery.

## Figures and Tables

**Table 1:** Comparison of existing solutions to the hand–eye calibration problem.

Categories	Solutions	Drawbacks
Separable solutions [10,11,12,13,14]	Solve the rotation part first; then, solve the translational part.	Error propagation problem.
Simultaneous solutions [15,16,17]	Solve the rotational and translational parts at the same time.	Sensitive to the nonlinearities present in measurements in the form of noise and errors.
Iterative solutions [8,18,19,20,21]	Solve a nonlinear optimization problem by minimizing the error by iteration.	Computationally expensive; may not always converge on the optimal solution.
Probabilistic methods [22,23]	Solve the calibration problem without the assumption of exact correspondence between the data streams.	Computationally expensive.

**Table 2:** Investigation of the influence of the range of robot movement to the hand–eye calibration.

	*d* [mm]		ϕ[°]	
*L* [mm]	Mean	Max.	Mean	Max.
30	1.17	1.40	0.87	1.25
60	0.86	1.09	0.83	0.93
90	0.82	0.95	0.72	0.91
120	0.86	1.06	0.75	0.90
150	0.71	1.11	0.70	0.85
200	0.70	0.88	0.68	0.96

**Table 3:** Comparison of our method with the SOTA hand–eye calibration methods.

	*d* [mm]		ϕ[°]		Computation Time [ms]
*L* [mm]	Mean	Max.	Mean	Max.	
Tsai [11]	0.74	0.92	0.75	0.88	1.18
Andreff [15]	0.73	0.87	0.70	0.92	2.23
Chou [13]	0.73	0.84	0.69	0.89	0.82
Shah [40]	0.74	0.92	0.72	0.97	0.63
Wu [8]	0.72	0.88	0.68	0.90	26.84
Ours	0.70	0.88	0.68	0.96	2.21

**Table 4:** Overall system accuracy study.

		Plastic Phantom	3D-Printed Vertebrae	Pig Vertebrae
*d* [mm]	Mean	0.70	0.66	0.71
	Max.	0.85	0.79	0.82
$d^{\prime }$ [mm]	Mean	0.93	0.90	1.01
	Max.	1.15	1.13	1.52
ϕ[°]	Mean	0.72	0.79	0.82
	Max.	0.94	0.91	0.96
$\phi^{\prime }\left[{}^{\circ}\right]$	Mean	1.04	0.96	1.11
	Max.	1.45	1.24	1.38

## Data Availability

Not applicable.

## Abbreviations

The following abbreviations are used in this manuscript:

CT	Computed Tomography
API	Application Programming Interface
COS	Coordinate System
SVD	Singular Value Decomposition
RHC	Registration-based Hand–eye Calibration
3D	Three-dimension
